# ZLM-7 exhibits anti-angiogenic effects via impaired endothelial cell function and blockade of VEGF/VEGFR-2 signaling

**DOI:** 10.18632/oncotarget.7968

**Published:** 2016-03-08

**Authors:** Min Su, Jingjia Huang, Jijia Li, Xiyuan Qin, Xiaoning Tang, Fang Jin, Shali Chen, Chuanming Jiang, Zizheng Zou, Kunjian Peng, Mohammed Nuruzzaman, Jianting Zhang, Junli Luo, Suyou Liu, Zhiyong Luo

**Affiliations:** ^1^ Molecular Biology Research Center, State Key Laboratory of Medical Genetics, School of Life Sciences, Central South University, Changsha 410078, China; ^2^ Department of Medicinal Chemistry, School of Pharmaceutical Sciences, Central South University, Changsha 410006, China

**Keywords:** angiogenesis, ZLM-7, microtubule, VEGF, VEGFR2

## Abstract

Inhibition of angiogenesis is a promising therapeutic strategy against cancer. In this study, we reported that ZLM-7, a combretastain A-4 (CA-4) derivative, exhibited anti-angiogenic activity *in vitro* and *in vivo*. *In vitro*, ZLM-7 induced microtubule cytoskeletal disassembly. It decreased VEGF-induced proliferation, migration, invasion and tube formation in endothelial cells, which are critical steps in angiogenesis. *In vivo*, ZLM-7 significantly inhibited neovascularization in a chicken chorioallantoic membrane (CAM) model and reduced the microvessel density in tumor tissues of MCF-7 xenograft mouse model. ZLM-7 also displayed comparable antiangiogenic and anti-tumor activities associated with the lead compound CA-4, but exhibited lower toxicity compared with CA-4. The anti-angiogenic effect of ZLM-7 was exerted via blockade of VEGF/VEGFR-2 signaling. ZLM-7 treatment suppressed the expression and secretion of VEGF in endothelial cells and MCF-7 cells under hypoxia. Further, ZLM-7 suppressed the VEGF-induced phosphorylation of VEGFR-2 and its downstream signaling mediators including activated AKT, MEK and ERK in endothelial cells. Overall, these results demonstrate that ZLM-7 exhibits anti-angiogenic activities by impairing endothelial cell function and blocking VEGF/VEGFR-2 signaling, suggesting that ZLM-7 might be a potential angiogenesis inhibitor.

## BACKGROUND

Solid tumors account for more than 90% of cancers depending on the vascular network [1, 2]. Strategies that disrupt pre-existing vasculature block the blood supply or inhibit specific steps in angiogenesis [3]. Individual agents might possess both vascular disrupting and anti-angiogenic activities [4]. It has been reported recently that microtubule-targeting agents (MTAs) are the most effective drugs exhibiting vascular-disrupting and anti-angiogenic activities [5].

Angiogenesis, the outgrowth of new capillaries from pre-existing blood vessels, is associated with the growth and progression of solid tumors [6]. Most solid tumors cannot grow beyond 1-2 mm^3^ in size without angiogenesis because the tissue oxygen diffusion limit is 100-200 μm [7]. Angiogenesis involves several steps, including degradation of the surrounding extracellular matrix, migration of endothelial cells, proliferation of endothelial cells and transformation of endothelial cells into tubular structures [8].

Of the nearly 30 known endogenous pro-angiogenic factors, the vascular endothelial growth factor (VEGF) is the predominant [9, 10]. VEGF stimulates numerous steps in tumor angiogenesis such as endothelial cell proliferation, migration and invasion, and is elevated in many solid tumors [11, 12]. Preclinical studies indicate that inhibition of VEGF signaling reduces tumor growth [13]. Vascular endothelial growth factor receptor 2 (VEGFR2) is the most important receptor in VEGF-induced angiogenesis [14]. VEGFR2 phosphorylation triggers downstream signaling cascade in endothelial cells [15–17]. Interruption of VEGF-VEGFR2 signaling pathway is a therapeutic target in tumor angiogenesis and solid tumor growth [18]. Disruption of microtubules may also downregulate VEGFR2 expression [19].

**Figure 1:**
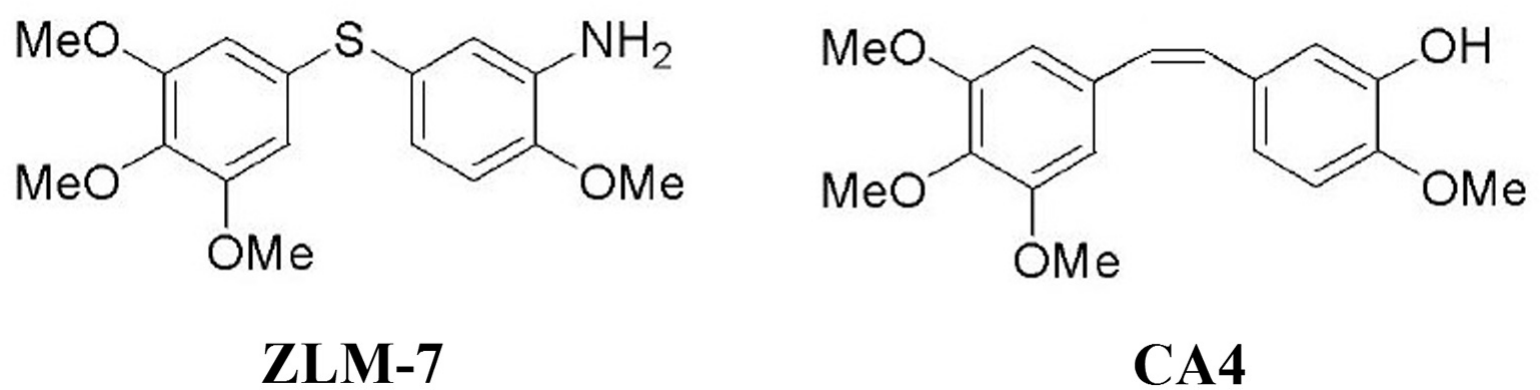
The chemical structure of ZLM-7 (molecular formula C_16_H_20_NO_4_S, molecular weight 322.4) and CA-4

One of the most potent MTAs is combretastatin A-4 (CA-4). CA-4 is currently undergoing clinical evaluation as a vascular disrupting agent [20]. It has strong affinity to tubulin leading to cytoskeletal destabilization [21]. However, the cis-configuration of CA-4 isomerizes to the more thermally stable trans-isomer, resulting in a dramatic decrease in activity [22]. Therefore, many derivatives with increased stability were designed and synthesized [23]. In a previous study, we developed a series of cis-restricted sulfide derivatives of CA-4, including ZLM-7 (Figure 1) exhibiting comparable anti-cancer activity to CA-4. In the present study, we evaluate the anti-angiogenic and anti-tumor activities of ZLM-7, and investigated its effect in VEGF-VEGFR2 signaling.

## RESULTS

### ZLM-7 disassembled microtubule network and induced G2/M phase arrest in HUVECs

**Figure 2:**
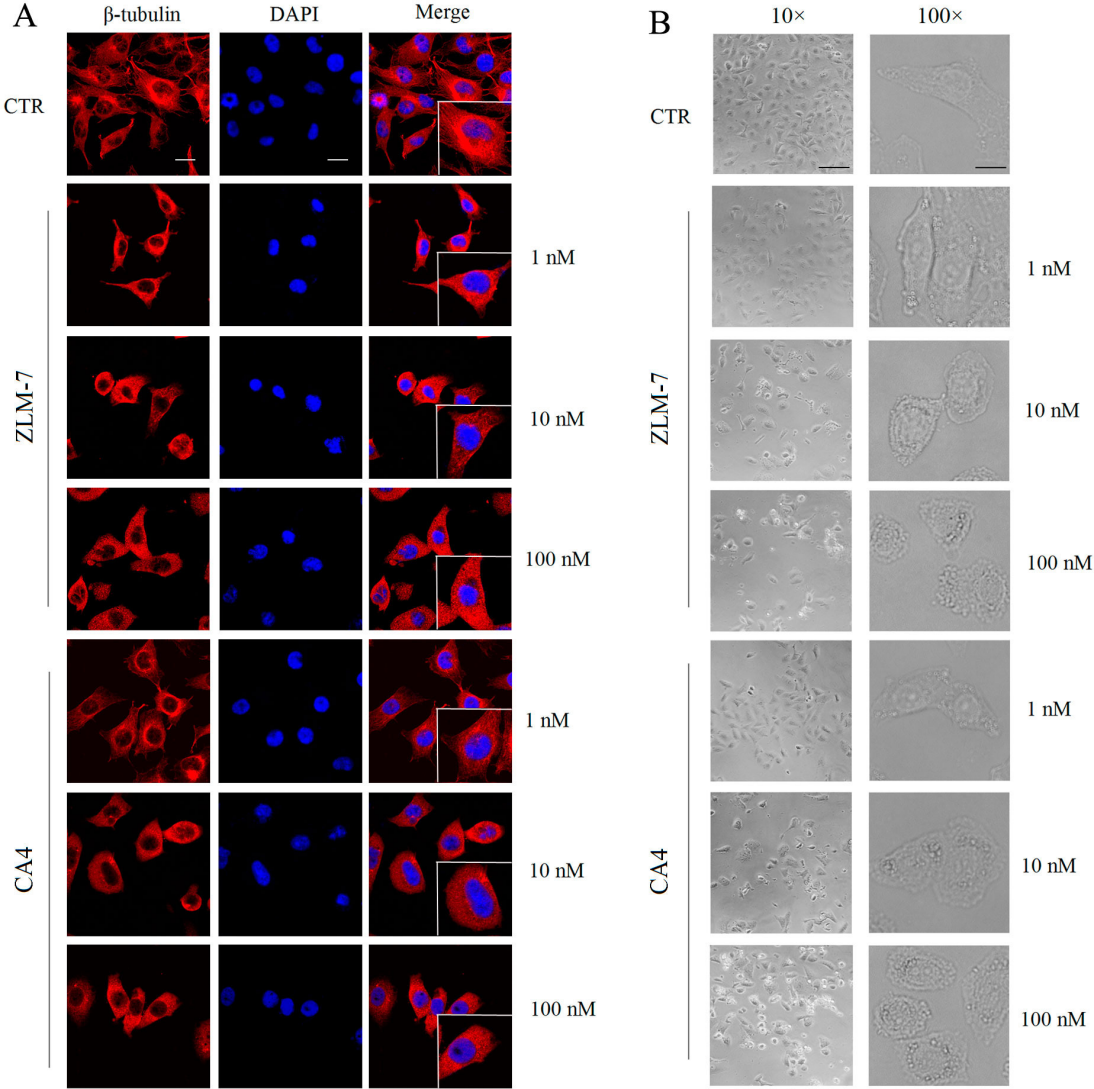
Effect of ZLM-7 on cytoskeleton and morphology in HUVECs **A.** Cells were treated for 24 h with ZLM-7 or CA-4, fixed and stained with anti-β-tubulin antibody, and photographed under confocal microscopy (63× magnification; bar = 10 μm). **B.** Cells were treated with or without ZLM-7 for 24 h and visualized by light microscopy at 10× (bar = 50 μm) or 100× (bar = 5 μm) magnification.

Human umbilical vein endothelial cells (HUVECs) play a key role in vascular sprouting. They are often used to investigate anti-angiogenic activity of drugs, *in vitro* [24, 25]. The microtubule network controls cell division and migration, maintenance of cell shape, intracellular trafficking of cell signaling in eukaryotic cells, and therefore, is required in angiogenesis [26]. To characterize the effect of ZLM-7 on microtubule cytoskeleton of HUVECs, immunofluorescent staining of β-tubulin was performed. Figure 2A showed that ZLM-7 treatment led to a diffuse microtubule network. In the control group, the microtubule network exhibited normal organization and arrangement of HUVECs. However, exposure to ZLM-7 (1-100 nM) caused microtubule disassembly and disappearance of microtubule polymerization at the cell periphery. The effects of the ZLM-7 on HUVEC morphology were also studied. We found that after 24 h of exposure to ZLM-7 at as low as 1 nM, cells became rounded, accompanied by membrane blebbing (Figure 2B). For comparison, we evaluated the effects of CA-4 under similar experimental conditions and found similar results with the microtubule network and morphology of HUVECs.

**Figure 3:**
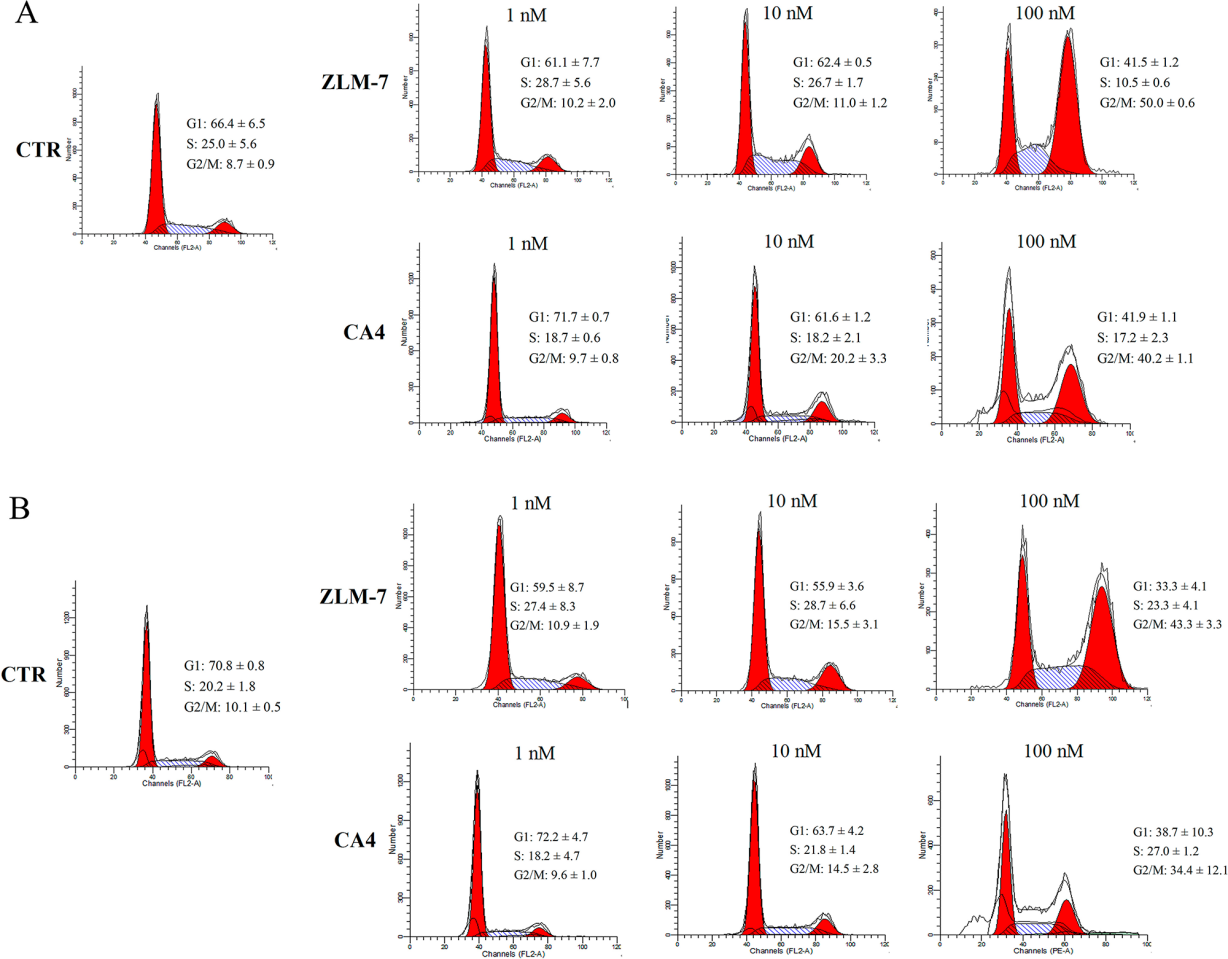
ZLM-7 caused cell cycle arrest at the G2/M-phase in HUVECs Cells were treated with ZLM-7 or CA-4 for **A.** 24 h or **B.** 48 h, and collected for cell cycle analyses via flow cytometry. All data were represented as means ± SD, n = 3.

Given the correlation between G2/M-phase arrest and tubulin polymerization [27], we examined the effect of ZLM-7 on the cell cycle by flow cytometry. Our data showed that ZLM-7 treatment induced a dose-dependent accumulation of cells in the G2/M-phase, with a reduction in the proportion of cells in G1-phase (Figure 3A and 3B). Similar results were obtained with CA-4.

### ZLM-7 reduced proliferation of HUVECs

**Figure 4:**
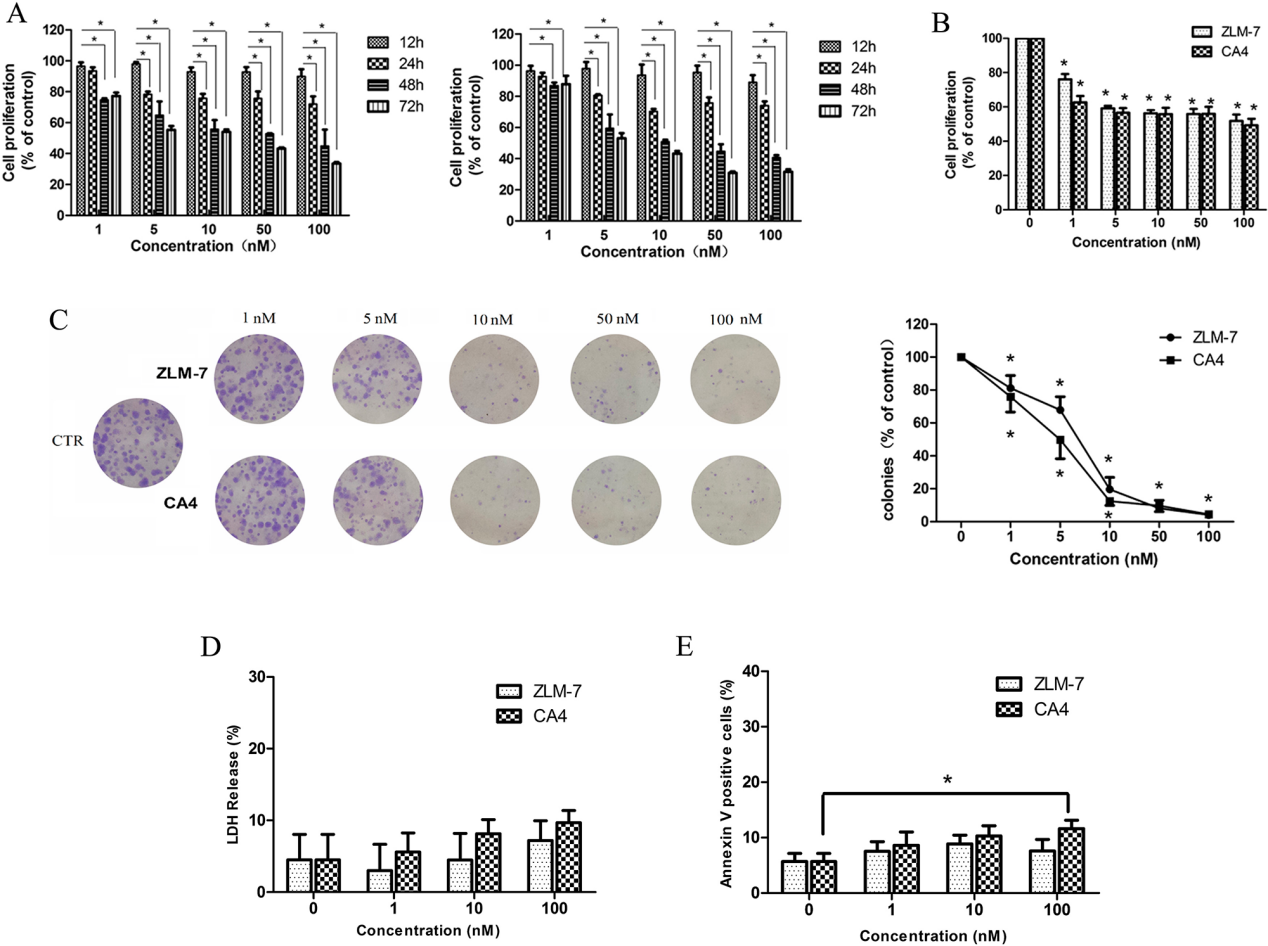
ZLM-7 inhibited HUVECs proliferation **A.** Cell proliferation was determined by MTT assay after treatment with ZLM-7 or CA-4 at varying times (12, 24, 48 and 72 h). **B.** Cell proliferation was determined by MTT assay after treatment with (left) ZLM-7 or (right) CA-4 for 48 h in the presence of VEGF. **C.** Representative images and quantitative data of the inhibitory effects of ZLM-7 and CA-4 in HUVEC clonogenic survival assay. **D.** Toxicity examination using LDH cytotoxicity assay of HUVECs after ZLM-7 or CA-4 treatment for 48 h. **E.** Annexin V-FITC analysis of apoptosis in HUVECs after treatment with ZLM-7 or CA-4 for 48 h by FACS. All data were represented as means ± SD, n = 3, * P < 0.05 compared to control.

Proliferation of HUVECs was detected by MTT assay to examine the anti-angiogenic effects of ZLM-7 *in vitro*. ZLM-7 and CA-4 inhibited the proliferation of HUVECs in a time- and concentration-dependent manner (Figure 4A). Approximately 50% reduction in cell number at the concentration of 10 nM after 48 h incubation was found relative to untreated cells. The results presented in Figure 4B showed that both ZLM-7 and CA-4 significantly suppressed VEGF-induced endothelial cell proliferation in a dose-dependent manner. The effect of ZLM-7 and CA-4 on HUVEC proliferation was similar.

To further evaluate the cytotoxicity of ZLM-7 on HUVECs, clonogenic assay, LDH cytotoxicity assay and Annexin V-FITC staining apoptosis assay were carried out. Results displayed in Figure 4C indicate that ZLM-7 was less toxic than CA-4 in the clonogenic assay. ZLM-7 was also found to induce fewer toxic effects than CA-4 on HUVECs visualized by LDH release (Figure 4D). As indicated in Figure 4E, the number of apoptostic cells was less in the ZLM-7-treated group compared with CA-4-treated group. The above results indicate that ZLM-7 exhibited lower cytotoxicity compared with CA-4 in HUVECs.

### ZLM-7 inhibited HUVECs migration, invasion and tube formation

**Figure 5:**
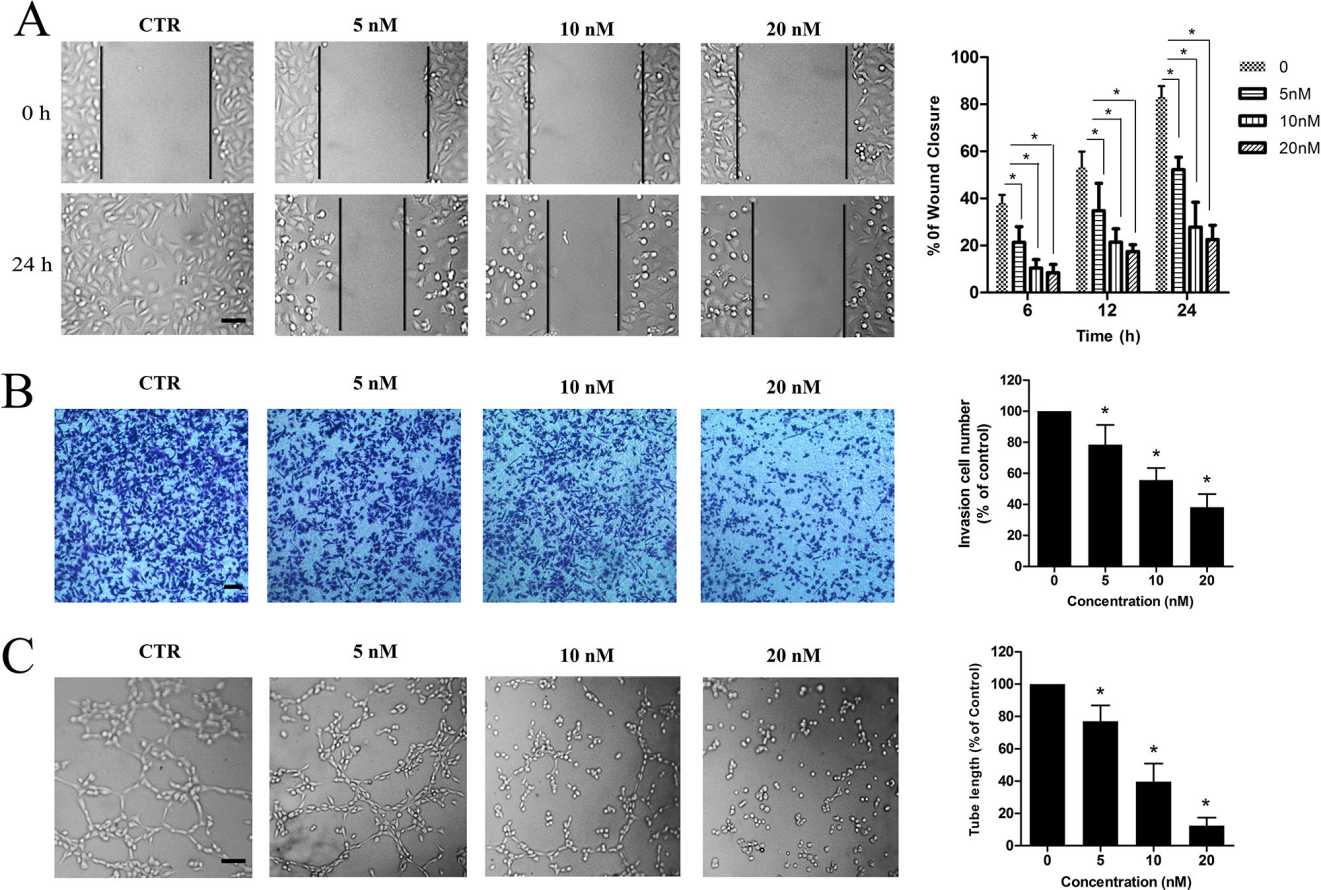
ZLM-7 suppressed VEGF-induced migration, invasion and tube formation of HUVECs **A.** Representative results and quantitative data of scratch wound-healing migration in cells treated with vehicle or ZLM-7 following VEGF stimulation. **B.** Representative results and quantitative data of Matrigel invasion assay of cells treated with vehicle or ZLM-7 for 24 h following VEGF stimulation. **C.** Representative results and quantitative data of tube formation of cells after vehicle or ZLM-7 treatment for 2h following VEGF stimulation. All images were 10 × magnification, bar = 20 μm. All data were represented as means ± SD, n = 3, * P < 0.05 compared with control.

Endothelial cell migration and invasion are key steps in angiogenesis [28]. To investigate the effect of ZLM-7 in endothelial cell migration, we used a wound-healing migration assay by mechanically scraping a clear space on HUVEC confluent monolayer for migration of motile cells. We observed that control cells almost completely migrated to fill in the initial clear area after 24 h, while ZLM-7 treatment significantly reduced the VEGF-induced migration of HUVECs in a concentration- and time-dependent manner (Figure 5A). In a Matrigel invasion assay, ZLM-7 also induced a dose-dependent suppression in the VEGF-induced invasion of HUVECs (Figure 5B). We further evaluated the inhibitory effects of ZLM-7 on endothelial tube formation, which is also a well known *in vitro* angiogenesis test [29]. As shown in Figure 5C, VEGF stimulation resulted in a rich network of branched capillary-like tubes 2 h after cells seeding on Matrigel. In the presence of ZLM-7, the capillary-like tubes were interrupted at lower concentrations (5 nM). Most cells formed spherical aggregates at higher concentrations (10, and 20 nM). In these experiments, we barely detected the difference between ZLM-7 and CA-4 (data not shown).

### ZLM-7 inhibited angiogenesis

**Figure 6:**
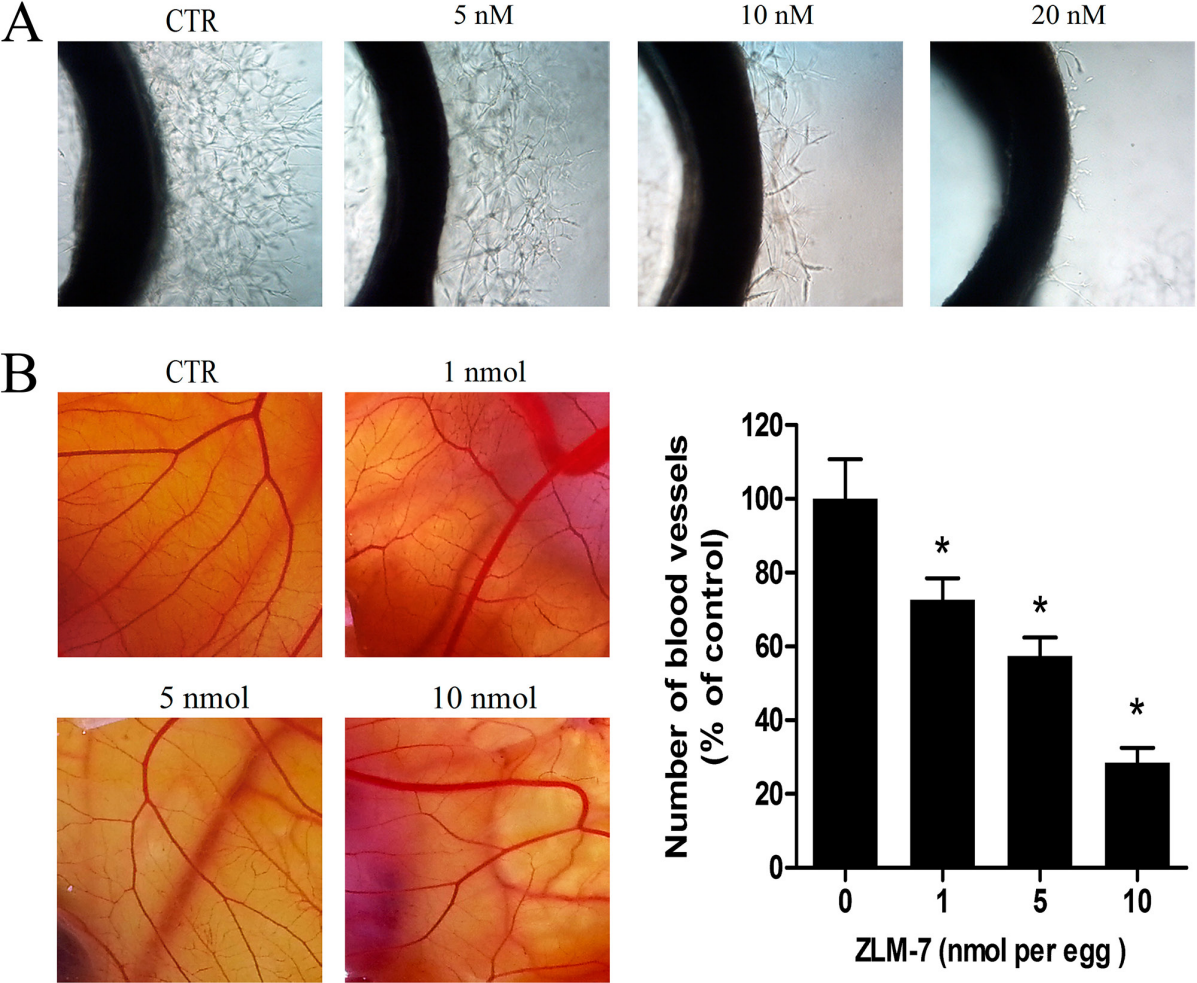
ZLM-7 inhibited angiogenesis **A.** Representative images of rat aorta sections after vehicle or ZLM-7 treatment. **B.** Representative images of CAM after vehicle or ZLM-7 treatment. Quantitative data were represented as means ± SD, n = 10, * P < 0.05 compared to control.

We evaluated the *ex vivo* anti-angiogenic activity of ZLM-7 based on capillary sprouting from aortic rings [30]. Microvessels emerging from cultured rat aorta embedded in Matrigel, mimic several stages of angiogenesis, including endothelial cell proliferation, migration and tube formation. As a result, the rat aortic ring assay simulates angiogenesis *in vivo*. The results showed that the control group formed a large number of microvessel structures in six-day-old cultures. However, 5 nM ZLM-7 notably suppressed the formation of microvessels and 20 nM ZLM-7 completely blocked microvessel sprouting from rat aortic rings (Figure 6A).

Next, we employed the popular CAM assay, which provides a unique *in vivo* model to investigate the anti-angiogenic effect of agents [31]. As shown in Figure 6B, normally developed CAMs in control were angiogenic, inducing a number of branches and new capillaries from the exiting basal vessels, whereas ZLM-7 blocked this angiogenesis. After 48 h of treatment with 5 and 10 nmol, ZLM-7 significantly impaired neovascularization accompanied by absence of vascular networks. Quantitative analysis revealed that 1, 5 and 10 nmol ZLM-7 caused 27.3%, 42.6% and 71.5% reduction in the number of blood vessels, respectively. These effects of ZLM-7 were similar to that of the lead compound CA-4 (data not shown).

### ZLM-7 inhibited hypoxia-induced VEGF and HIF-1α expression

**Figure 7:**
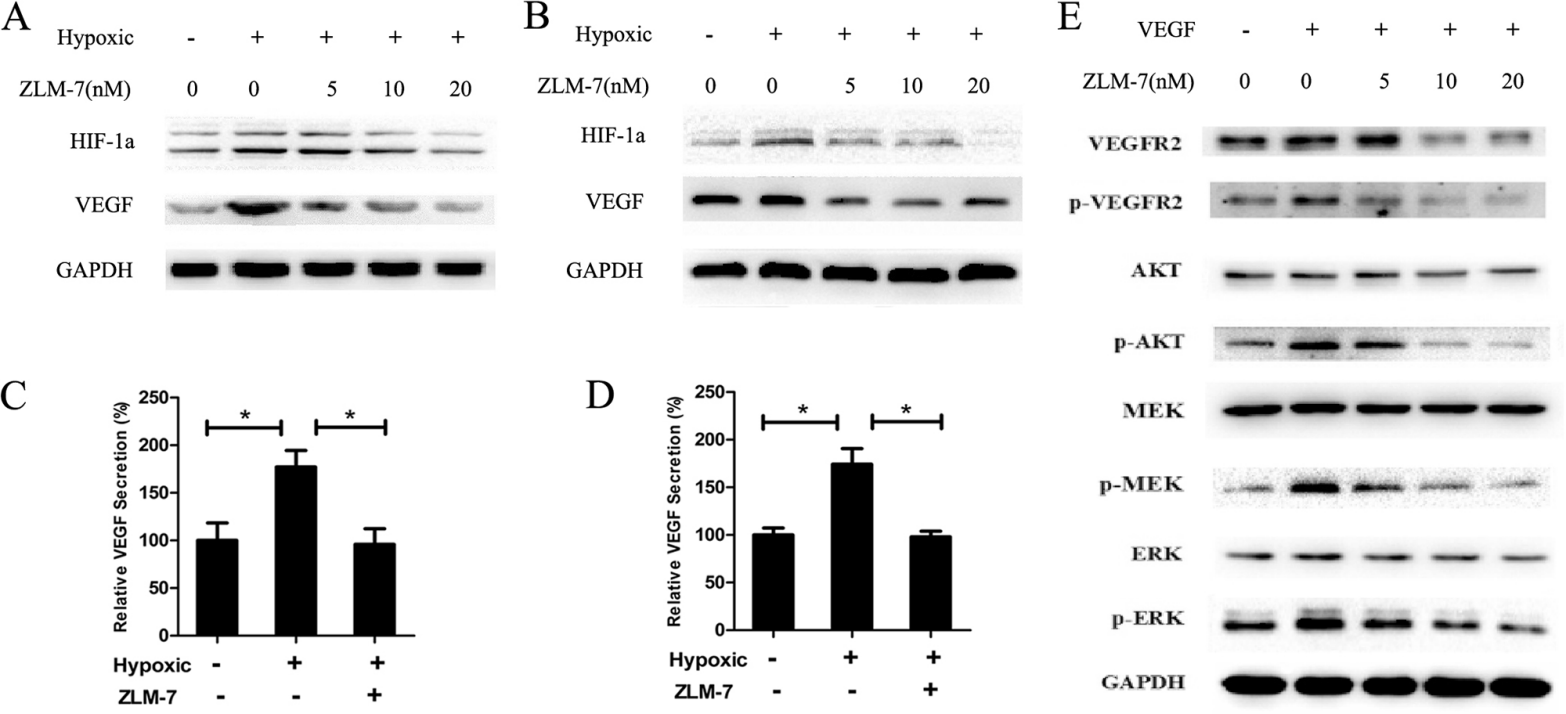
ZLM-7 down-regulated VEGF-VEGFR2 signaling pathway A representative result of Western blot showed the levels of VEGF and HIF-1α in **A.** HUVECs and **B.** MCF-7 cells incubated with vehicle or ZLM-7 for 24 h at normoxia, or treated for 12 h at normoxia and then subjected to an additional 12 h of hypoxia. Secretion of VEGF in medium was measured by ELISA in **C.** HUVECs and **D.** MCF-7 cells treated with vehicle or 10 nM ZLM-7 as described in (A). Data were represented as means ± SD, n = 3, * P < 0.05 compared with control. **E.** A representative Western blot showed the levels of total or phosphorylated form of VEGFR-2, AKT, MEK and ERK in HUVECs treated with vehicle or ZLM-7 for 24 h with or without VEGF stimulation.

VEGF is a pro-angiogenic factor induced by hypoxia-inducible factor-1α (HIF-1α), under hypoxic conditions [9, 32]. We investigated the effects of ZLM-7 on VEGF and HIF-1α protein expression under hypoxic conditions for 12 h by Western blot. Our data revealed that both in HUVECs and MCF-7 cells, the amount of VEGF and HIF-1αprotein increased under hypoxia compared with normoxic conditions. ZLM-7 treatment decreased protein expression in a dose-dependent manner (Figure 7A and 7B). The secretion of VEGF was also increased in HUVECs and MCF-7 cells in hypoxia, whereas it was dramatically decreased after ZLM-7 treatment (Figure 7C and 7D).

### ZLM-7 suppressed VEGFR2 activation and its downstream signaling pathways

VEGF induces angiogenesis by stimulating the proliferation, migration and sprouting of endothelial cells via binding to VEGFR2 [15]. We further evaluated the effects of ZLM-7 on the VEGF2 and its downstream signaling pathways. As shown in Figure 7E, the total VEGFR2 expression level decreased upon treatment with ZLM-7. VEGF stimulated VEGFR2 phosphorylation, which was inhibited by ZLM-7. VEGF also activated VEGFR2 downstream signaling molecules including AKT, ERK1/2, and MEK, which was inhibited by ZLM-7 in a dose-dependent manner. However, the total expression of these proteins was almost unaffected.

### ZLM-7 inhibited tumor growth and angiogenesis in a MCF-7 mouse xenograft model

**Figure 8:**
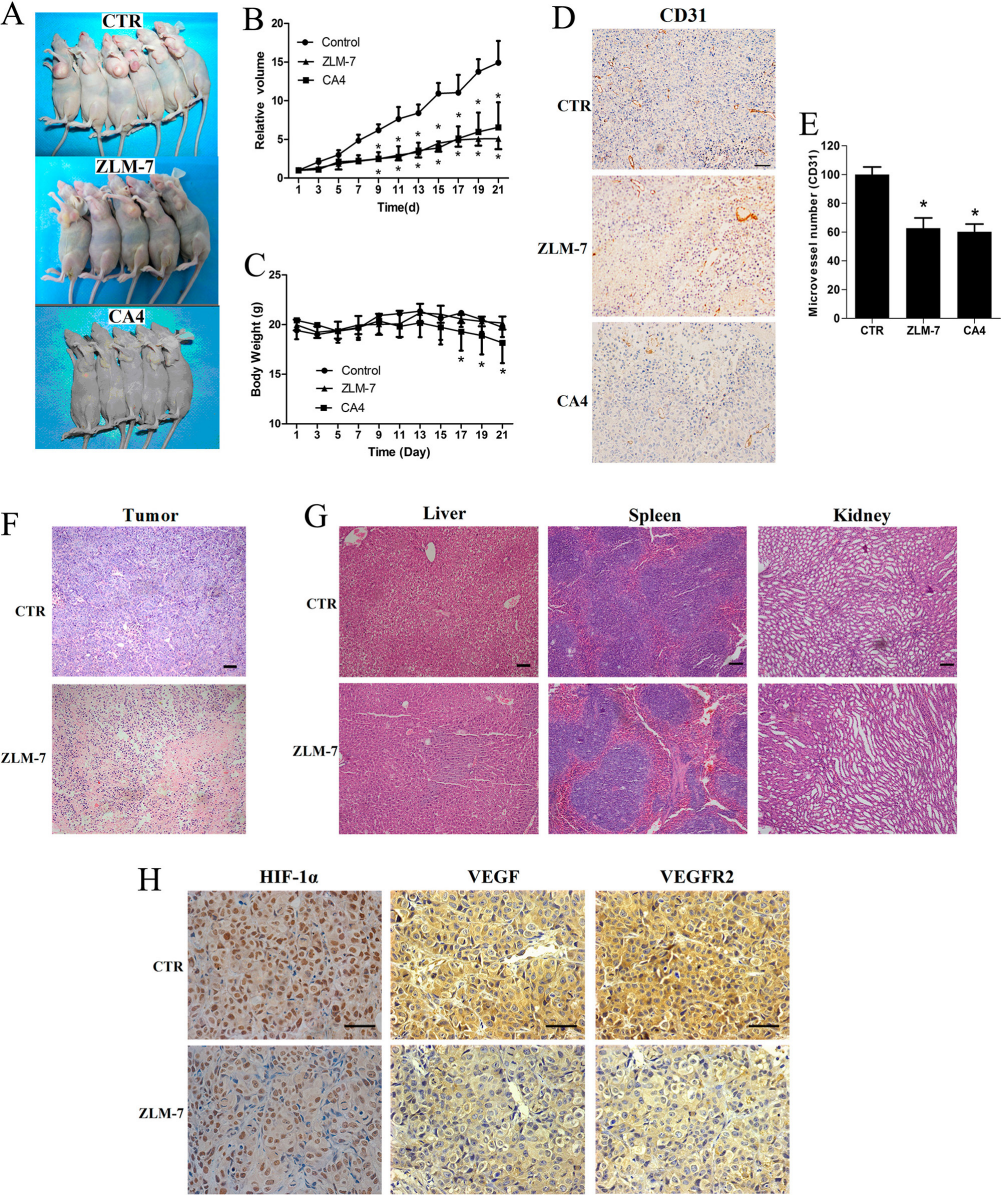
ZLM-7 inhibited tumor growth and neoangiogenesis in MCF-7 breast cancer xenografts Animals were treated with 15 mg/kg ZLM-7 or CA-4 daily. **A.** Anti-tumor effects of ZLM-7 and CA-4 on MCF-7 bearing mice. **B.** *In vivo* tumor growth inhibition activity of ZLM-7 and CA-4. **C.** Change in body weight of mice. **D.** CD31 immunohistochemistry staining tumor tissues (bar = 20 μm). **E.** Quantitative analysis of tumor section stained with CD31. **F.** HE staining of tumor tissues (10×, bar = 20 μm). **G.** HE staining of liver, spleen and kidney (10×, bar = 20 μm). **H.** Immunohistochemical detection with specific antibodies against HIF-1α, VEGFA and VEGFR2 (40×, bar = 20 μm). Data were represented as means ± SD, n=5, *P < 0.05 versus control.

To elucidate the anti-tumor effects of ZLM-7 *in vivo*, we used an MCF-7 breast xenograft mouse model. In this experiment, 15 mg/kg ZLM-7 or CA-4 was administrated i.p. daily for 3 weeks. The results showed that ZLM-7 therapy significantly suppressed the growth of MCF-7 subcutaneous tumor (Figure 8A and 8B). The relative tumor volume in ZLM-7 and CA-4 group decreased 65.8% and 56.0%, respectively. Meanwhile, ZLM-7 treatment did not significantly decrease the mouse body weight compared with control (P > 0.05) (Figure 8C). Further, CA-4 significantly decreased the body weight (P < 0.05), which suggested that ZLM-7 was less toxic than CA-4.

We further investigated whether ZLM-7 inhibited angiogenesis in tumor, via immunohistochemistry with anti-CD31 antibody to stain solid tumor sections. CD31 is present on most capillaries and is a widely used endothelial marker for quantifying angiogenesis [33]. ZLM-7 treatment markedly reduced the development of newly formed vessels in tumors (Figure 8D). The mean vessel density in tumors treated with ZLM-7 and CA-4 was reduced to 62.7% and 60.2%, respectively (Figure 8E).

As shown in Figure 8F, H&E staining revealed that ZLM-7 therapy significantly elevated tumor necrosis. In addition, ZLM-7 induced no apparent pathological abnormalities in normal tissues including liver, spleen and kidney (Figure 8G). Consistent with our *in vitro* data, ZLM-7 treatment strongly decreased the HIF-1α, VEGF and VEGFR2 protein expression levels (Figure 8H).

## DISCUSSION

CA-4 is one of the latest additions to the anticancer drug candidates undergoing phase III clinical trials. Previous structure–activity relationship studies (SAR) of CA-4 confirmed that the cis-orientation of the diaryl groups was essential for its strong cytotoxicity. However, this compound is intrinsically unstable due the isomerisation of the cis-isomer into a more thermodynamically stable but inactive trans-isomer [34]. To circumvent the problem of cis-trans isomerization, chemically stable cis-restricted derivatives of CA-4 were obtained by incorporating the olefinic double bond with one-atom or five-member aromatic heterocyclic rings [35, 36]. In our previous studies, we identified ZLM-7 as the most active compound among a series of sulfide derivatives of CA-4. In this study, we unraveled the anti-angiogenic and anti-tumor activities of ZLM-7.

We found that ZLM-7 inhibited angiogenesis both *in vitro* and *in vivo*. ZLM-7, at nanomolar concentrations, dramatically inhibited angiogenesis-associated activities including proliferation with or without stimulation by VEGF, VEGF-stimulated migration, invasion and vessel-like tube formation of endothelial cells. In addition, in an *ex vivo* rat aortic arch model, the sprouting of ZLM-7-treated aorta was scattered compared with the control. Nevertheless, the above *in vitro* and *ex vivo* models lack the biological complexity of vascular system in vertebrates. Evidence supporting *in vivo* anti-angiogenic effects of ZLM-7 is derived from the chicken chorioallantoic membrane (CAM) and the MCF-7 xenograft mouse model. ZLM-7 obviously reduced the neovascularization on CAM and significantly suppressed tumor growth accompanied by reduced microvessel density (MVD) in tumor tissues of the MCF-7 xenograft model. ZLM-7 also induced minor toxic effects in endothelial cells at concentrations that significantly inhibited angiogenesis *in vitro*. In addition, no body weight loss or toxicity was observed in normal tissues of the ZLM-7 treated mouse xenograft model. Interestingly, ZLM-7 showed similar antiangiogenic and anti-tumor activities with the lead compound CA-4, whereas it exhibited lower toxicity compared with CA-4. These results indicated that ZLM-7 may be an angiogenesis inhibitor with limited toxicity.

Microtubule cytoskeleton controls cell cycle progression, cell proliferation and cell migration [26, 37]. In this study, we found that ZLM-7 disrupted the microtubule cytoskeleton of endothelial cells. Inhibition of microtuble polymerization resulted in cell cycle arrest in various cell lines [27]. We also found that ZLM-7 induced G2/M-phase arrest in endothelial cells. These findings strongly suggested that the morphological changes caused by ZLM-7 might be correlated with endothelial function in angiogenesis.

Microtubules also affect signal transduction pathways in cells [38]. We investigated the intrinsic molecular mechanisms underlying the anti-angiogenic activities of ZLM-7. In tumors, the microenvironment is predominantly hypoxic [39]. Tumor growth is induced via transcription and secretion of pro-angiogenic factors controlled by hypoxia-inducible factor (HIF) resulting in an angiogenic phenotype [40]. VEGF expression is induced by HIF-1 in response to hypoxia [41, 42]. In this study, we found that ZLM-7 suppressed HIF-1α expression, and VEGF expression and secretion in HUVECs and MCF-7 cells under hypoxia. The results are consistent with the results of microtubule disrupting drugs, which inhibit tumor angiogenesis via the HIF-1 pathway [43–45]. These results suggested that ZLM-7 might inhibit tumor angiogenesis via paracrine and autocrine VEGF pathways.

The angiogenic response to VEGF occurs mainly via activation of VEGFR2 [46, 47]. In the present study, we demonstrated that ZLM-7 suppressed the expression of total and phosphorylated VEGFR2 stimulated by VEGF. ZLM-7 also inhibits multiple VEGFR2 downstream signaling mediators such as activated AKT, MEK and ERK. AKT is responsible for the proliferation and survival of endothelial cells; MEK and ERK activation results in increased proliferation and migration of endothelial cells [9]. In addition, the findings *in vivo* were consistent with our *in vitro* results, indicating that the reduced MVD in tumors may be related to ZLM-7 suppression of the expression of HIF-1α, VEGF and VEGFR-2. All these results suggested that ZLM-7 treatment inhibited tumor angiogenesis by suppression of the VEGF-VEGFR2 signaling pathway.

In conclusion, our data indicate that ZLM-7 exerts similar antiangiogenic and anti-tumor effects with low toxicity compared with CA-4. ZLM-7 represents a promising candidate as angiogenesis inhibitor.

## MATERIALS AND METHODS

### Reagents, cells and animals

Anti-VEGFR2, Anti-phosph-VEGFR2 (T1175), anti-AKT, anti-phosph-AKT (S473), anti-ERK-1/2, anti-phosph-ERK-1/2(T202/Y204), anti-MEK, anti-phosph-MEK (S218/S222), anti-DAPDH antibodies were purchased from Cell Signaling Technology (Danvers, MA, USA). Anti-VEGF, anti-HIF-1α and anti-CD31 antibodies were purchased from Abcam (Louis Park, MN, USA). Anti-β-tubulin antibody was ordered from Sigma-Aldrich (Milano, Italy). Medium M199, Dulbecco's modified Eagle's medium (DMEM), and fetal bovine serum (FBS) were supplied by Hyclone Laboratories (South Logan, Utah, USA). Vascular endothelial growth factor (VEGF) was obtained from PeproTech (Rocky Hill, NJ, USA). Matrigel were purchased from BD Bioscience (Bedford, MA, USA). ZLM-7 was synthesized as previously described [48]. CA-4 was synthesized as described [21, 49]. Stock solutions were prepared in dimethyl sulfoxide (DMSO as vehicle) and stored in aliquots at −20°C.

HUVECs were obtained from Xiangya Central Experiment Laboratory as previously described [50]. The human breast carcinoma cell line MCF-7 was purchased from the Cell Bank of the Chinese Academy of Sciences (Shanghai, China). HUVECs and MCF-7 cells were grown in M199 medium and DMEM medium supplemented with 10 % FBS, respectively. To maintain normoxic conditions, cells were incubated at 37°C in a 5 % CO_2_ atmosphere. To induce hypoxia, cells were incubated in an anaerobic chamber at 5 % CO_2_ with 1 % O_2_ balanced with N_2_.

Sprague-Dawley rats and Balb/c nu/nu mice were obtained from Laboratory Animal Center of Central South University (Changsha, China). All procedures involving animals were performed in compliance with the Guide for the Care and Use of Laboratory Animals of Central South University, with the approval of the Scientific Investigation Board of Central South University.

### Cytoskeletal immunofluorescence

HUVECs were plated on glass coverslips and allowed to attach for 12 h before ZLM-7 or CA-4 was added for an additional 24 h. Cells were fixed with cold 4% paraformaldehyde for 15 min, permeabilized with 0.1 % Triton X-100/TBS for 10 min and blocked with 5 % BSA for 1 h. The cells were then incubated overnight with primary anti-β-tubulin antibody at 4°C and an Alexa-conjugated secondary antibody (Life technologies, Monza, Italy) for 1 h. Cells were counterstained with DAPI. Fluorescent images were obtained using a confocal microscope (Vico, Ecliple Ti80, Nikon).

### HUVEC morphology

HUVECs were plated at in 6-well plates at an optimal counting density and incubated overnight for attachment. The cells were then treated with ZLM-7 or CA-4. After 24 h, the cell morphology was monitored microscopically and photographed.

### Cell cycle analysis

HUVECs were seeded in 100-mm dishes. After 12 h incubation for attachment, ZLM-7 or CA-4 was added and cells were incubated for another 24 h or 48 h. Then, both the floating and adhesive cells were collected and fixed with ice-cold 75 % ethanol (4°C, overnight). After ethanol removal, the cells were stained with a cell cycle analysis kit (containing 1 mg/mL PI and 10 mg/mL RNase A) for 30 min at room temperature in the dark. Cell cycle distribution was determined using a FACScan flow cytometer (BD FACS Calibur).

### Cell proliferation assay

HUVECs in 96-well plates were allowed to adhere overnight followed by addition of ZLM-7 or CA-4 within a concentration range of 1 to 100 nM in the presence or absence of VEGF (20 ng/mL). After appropriate incubation, the cell proliferation was measured using a 3-(4, 5-dimethyl-2-thiazolyl)-2, 5-diphenyl-2H-tetrazolium bromide (MTT) colorimetric assay.

### Colony forming assay

HUVECs plated in 6-well plates were allowed to adhere for 12h. After treatment with different concentrations of ZLM-7 or CA-4 for 4 h, the medium was refreshed and cells were cultured for 2 weeks to obtain colonies (replacing culture medium every 2 days). Subsequently, the cells were briefly washed with PBS, stained with crystal violet and photographed. Colonies of >50 cells were counted visually.

### LDH toxicity assay

The cytotoxicity of ZLM-7 was determined by measuring the release of lactate dehydrogenase (LDH) activity into the medium. HUVECs were seeded in 96-well plates and allowed to adhere for 12h. After incubation with ZLM-7 or CA-4 for 48 h, cell supernatants were collected and analyzed for LDH activity using LDH cytotoxicity assay kit from Beyotime (Shanghai, China).

### Apoptosis assay

The effects of ZLM-7 on cellular apoptosis were analyzed using Annexin V-FITC/PI Apoptosis Detection Kit. After incubation overnight, HUVECs were treated with ZLM-7 or CA-4 for 48 h. The cells were stained with Annexin V-FITC and propidium iodide (PI) for 15 min. The apoptosis was analyzed by flow cytometry (BD FACS Calibur).

### Motility assay

HUVECs were seeded in 6-well plates. After a confluent monolayer was formed, the cells were mechanically wounded using a pipette tip. The wells were then rinsed with PBS to remove the dislodged cells and added M199 medium with 1% FBS and VEGF (20 ng/mL) containing vehicle or ZLM-7. At indicated time points following the drug addition, the cells were photographed under a light microscope. The wound width was measured and the percent of gap closure was calculated.

### *In vitro* chemoinvasion assay

A 24-well plate transwell chamber with 8.0 μm pore sized polycarbonate filter inserts from Corning Costar (Coring, NY, USA) was used. The upper side was coated with 30 μL Matrigel. After 1 × 10^5^ HUVECs were placed in M199 medium containing 1% FBS and vehicle or ZLM-7 in the upper compartment of the filter, the lower chamber was filled with medium M199 (1 % FBS) containing 20 ng/mL VEGF as a chemoattractant. The chamber was incubated at 37°C for 24 h. The cells on the lower side of the filter membrane were then fixed with 4% paraformaldehyde, stained with crystal violet and photographed microscopically.

### Tube formation assay

We seeded 2×10^4^ HUVECs in complete medium containing vehicle or ZLM-7 and 20 ng/mL VEGF on polymerized Matrigel, in 96-well plates. After incubation for 2 h, the cells were photographed under microscopy. The effect of the drugs on capillary tube formation was calculated by measuring the length of cell network.

### Aortic ring assay

The 48-well plates were covered with 100 μl Matrigel. Aortic rings obtained from 6-week-old male Sprague-Dawley rats were rinsed five times with the M199 medium, sectioned into 1 mm-long cross sections and placed on the surface of Matrigel in wells and covered with an additional 60 μl Matrigel. The rings were cultured in 0.4 mL M199 medium containing 20% FBS and either vehicle or various concentration of ZLM-7. Each group contained six aortic rings. The medium was replaced every 2 days. After 6 days of incubation at 37°C, the microvessel growth was photographed microscopically.

### CAM assay

Groups of 10 fertilized chicken eggs were incubated at 37.8°C and 60–70 % relative humidity for 8 days. The chicken chorioallantoic membrane (CAM) was separated from the shell membrane by drilling a small hole on the broad end of the egg and another hole at a position 90° from the first. A 1 cm^2^ window was removed from the eggshell immediately over the second hole and the membrane was carefully pushed down to detach the CAM from the shell. Sterilized filter paper disk (5 × 5 mm) saturated with either vehicle or ZLM-7 (1, 5 or 10 nmoL/egg) was placed on the CAM. The windows were sealed with cellophane tape and the eggs were further incubated for 48 h. After incubation, the papers were removed and the CAMs were photographed. Angiogenesis was quantified by counting the number of blood vessel branch points in each photo.

### Western blot analysis

HUVECs or MCF-7 cells were incubated in the presence or absence of ZLM-7 under indicated conditions and were then collected. Total protein extracts were obtained by lysing cells in cold RIPA buffer containing a proteinase inhibitor and a phosphatase inhibitor (Sigma-Aldrich, St. Louis, MO) and centrifuged at 15,000 g for 20 min at 4°C. The cell lysates were separated by 10% sodium dodecyl sulfate-polyacrylamide gel electrophoresis (SDS-PAGE) and transferred to polyvinylidene difluoride (PVDF) membranes. Membranes were blocked and immunolabeled overnight at 4°C with primary antibodies against VEGF, HIF-1α, GAPDH, total and phosphorylated VEGFR2, AKT, ERK and MEK. The membranes were then incubated with secondary antibodies. Immunolabeling was visualized using the enhanced chemiluminescence (ECL) detection system (GE healthcare) according to the manufacturer's instructions.

### ELISA assay

VEGF secretion was measured in HUVECs and MCF-7 cells using a VEGF immunoassay kit from Research & Diagnostics Systems, Inc. (Minneapolis, MN, USA). Cells were incubated with or without 10 nM ZLM-7 for 24 h under indicated conditions. After treatment, the supernatants were collected by centrifugation at 5,000 rpm for 20 min at 4°C. VEGF protein levels were determined according to the manufacturer's protocol.

### Xenograft models and immunohistochemistry analysis

1 × 10^7^ MCF-7 cells were injected subcutaneously into the right flank of female Balb/c nu/nu mice. Mice were randomly divided into two groups (n = 5) and treated intraperitoneally with ZLM-7 or CA-4 (15 mg/kg/day) when the tumors reached about 100 mm^3^. The body weight and tumor size of each mouse every other day was recorded. Solid tumor volume (V) was determined by measuring the longest diameter (A) and shortest diameter (B) of the tumor using digital vernier caliper measurements and calculated as follows: V = (A × B^2^)/2. After sacrificing mice on day 21, the tumors and normal tissues were harvested for molecular assessment.

Excised tumors and normal tissues were fixed in 10% buffered formalin solution and embedded in paraffin. Histological assessment was conducted by staining with hematoxylin and eosin (H&E) to investigate the induction of necrosis and analyze the toxicity. The tumor sections were immunohistochemically stained with anti-CD31, anti-HIF-1α, anti-VEGF and anti-VEGFR2 antibodies. Microvessels stained with CD31 were quantified in five arbitrarily selected fields from each tumor.

### Statistical analysis

The data are expressed as mean ± standard deviation (SD). Statistical analysis was conducted using SPSS 16.0 software. The Student's t-test was used between two groups and the differences were considered significant at P-values < 0.05.
